# Clinical and Radiological Features of Adult Onset Bilateral Medial Frontal Cerebral Cortical Encephalitis With Anti-myelin Oligodendrocyte Glycoprotein Antibody

**DOI:** 10.3389/fneur.2020.600169

**Published:** 2020-12-16

**Authors:** Juichi Fujimori, Masashi Nakamura, Takahito Yagihashi, Ichiro Nakashima

**Affiliations:** Division of Neurology, Tohoku Medical and Pharmaceutical University, Sendai, Japan

**Keywords:** MRI, anterior cerebral artery, bilateral medial frontal lobe, myelin oligodendrocyte glycoprotein, cerebral cortical encephalitis

## Abstract

**Objective:** To clarify the clinical and radiological features of adult onset anti-myelin oligodendrocyte glycoprotein (MOG) antibody-associated bilateral medial frontal cerebral cortical encephalitis (BFCCE).

**Methods:** We systematically reviewed the literature for patients with anti-MOG antibody-associated BFCCE. Patients who were also positive for other encephalitis-related autoantibodies were excluded from the study. The frequency of several characteristic neurological symptoms and lesion distributions were analyzed.

**Results:** We identified six patients with anti-MOG antibody-associated BFCCE. Among them, 6/6 had headache, 4/6 had fever, 3/6 had seizure, 2/6 had paraparesis, 2/6 had lethargy, and 2/6 had memory disturbance. CSF pleocytosis was observed in 5/6 patients, while CSF myelin basic protein was not elevated in any of the six patients. On brain MRI, 6/6 had bilateral medial frontal cortical lesions, 3/6 had corpus callosum lesions, and 3/6 had leptomeningeal enhancements. Most of the lesions were distributed in the territory of the anterior cerebral artery (ACA).

**Conclusion:** Our results indicate that anti-MOG antibody-associated BFCCE presents with characteristic clinical symptoms and MRI findings, which might reflect lesion formation in the ACA territory.

## Introduction

Anti-myelin oligodendrocyte glycoprotein (MOG) antibody-associated disease is a recently established spectrum of diseases. Conformation-sensitive antibodies against MOG are detectable in patients with optic neuritis, myelitis, opticomyelitis, acute or multiphasic disseminated encephalomyelitis (ADEM/MDEM) and brainstem/cerebral cortical encephalitis, although they are rarely detected in patients with prototypic multiple sclerosis or anti-aquaporin 4 (AQP4) antibody-positive neuromyelitis optica spectrum disorders (NMOSDs) (1–13).

Cerebral cortical encephalitis with MOG antibody is a disease entity that we first described and can be divided into two types (9, 10). One is unilateral (9), and the other is the bilateral medial frontal type (10). The unilateral type was first reported as part of a case series of four patients (9). All the patients were adults, and their main symptom was focal seizures, which often evolved to bilateral tonic-clonic seizures. Cerebrospinal fluid (CSF) pleocytosis was observed in these patients, and brain MRI demonstrated unilateral cerebral cortical hyperintensities best seen on fluid-attenuated inversion recovery (FLAIR) sequence, which were swollen and corresponded to hyperperfusion on single photon emission computed tomography (SPECT). Subsequent to these reports, similar cases have been reported that have broadened the clinical spectrum of this disorder (11, 14–17). Meanwhile, the bilateral medial frontal type was reported in a case report in which an adult patient also presented with secondary generalized seizure, CSF pleocytosis, and bilateral cerebral cortical hyperintensities best seen on FLAIR sequence (10).

According to a recent study by Wang et al., 20.7% (18/87) of anti-MOG antibody-positive patients presented with encephalitis, while six out of 18 were also positive for CSF anti-N-methyl-D-aspartate (NMDA) receptor (NMDAR) antibodies, and 94.4% (17/18) had favorable outcomes. Furthermore, 28% (5/18) of patients with anti-MOG antibody-associated encephalitis had unilateral cerebral cortical lesions, and 33% (6/18) had frontal and/or parietal cortical lesions close to the cerebral falx (16). In contrast, Cobo-Calvo et al. reported abnormal brain MRI in 44% (49/108) of patients with anti-MOG antibody-associated diseases. Among them, unilateral and bilateral cerebral cortical lesions were found in 10% (5/49) and 6% (3/49) of patients, respectively (18).

Although cases of unilateral cerebral cortical encephalitis (UCCE) with anti-MOG antibody have accumulated and were recently reported in a review article (14), few cases of bilateral medial frontal cerebral cortical encephalitis (BFCCE) with anti-MOG antibody have been reported in the literature (16, 18). Therefore, to clarify the clinical and radiological features of adult-onset anti-MOG antibody-associated BFCCE, we conducted a literature search focusing on this disease.

## Methods

We reviewed the literature for cases of anti-MOG antibody-associated BFCCE. We searched PubMed and the literature in Clinical and Experimental Neuroimmunology (CENI), which is an international journal sponsored by the Japanese Society for Neuroimmunology (https://onlinelibrary.wiley.com/journal/17591961), for “[bilateral] AND [MOG],” “[bilateral] AND [encephalitis],” “[MOG] AND [encephalitis].” All relevant published articles dating back 10 years from August 13, 2020 were reviewed for potential study inclusion. Patients were included if they (a) were older than 17 years, (b) had predominantly bilateral medial frontal cortical T2-FLAIR hyperintensities at presentation and (c) had MOG-IgG antibodies that were identified by cell-based assay (CBA) in serum and/or CSF. Patients were excluded if (a) they were copositive for other encephalitis-related autoantibodies and (b) lacked sufficient data. A total of 1,051 search results were screened for potential inclusion in this review.

To better visualize the distributions of cortical and juxtacortical lesions in previously reported cases of BFCCE and UCCE with anti-MOG antibodies on MRI, we superimposed them with a computer software program (PowerPoint 2016). Transparencies of the traced lesions on axial FLAIR images were overlaid on standardized axial sections (at the level of the lateral ventricles and the centrum semiovale) so that the densities reflected the distribution of the lesions.

## Results

We identified six adult patients with anti-MOG antibody-associated BFCCE (Table 1). The mean age was 34 years (range 18–46 years), and 3/6 (50%) were male. Five patients were Japanese, and the other was Chinese. Among them, 6/6 had headache, 4/6 had fever, 3/6 had seizures (secondary generalized seizures in two patients, epilepsia partialis continua in one patient), 2/6 had paraparesis, 2/6 had lethargy, and 2/6 had memory disturbance. On brain MRI, 6/6 had bilateral medial frontal cortical lesions, 3/6 had corpus callosum lesions, and 3/6 had leptomeningeal enhancements.

**Table 1:** Previously reported adult cases with anti-MOG antibody associated bilateral medial frontal cortical encephalitis.

			**Clinical symptoms during the course of illness**						**Brain MRI findings**			**CSF findings**		**Treatment**
	**Publication**	**Age/Sex**	**Headache**	**Fever**	**Seizure**	**Paraparesis**	**Memory disturbance**	**Lethargy**	**Bilateral frontal cortical lesion**	**Corpus callosum lesion**	**Gd enhance-ment**	**CSF cells**	**CSF MBP (<102 pg/ml)**	**Steroid responsive-ness**
Case 1	Kamada et al.	32/F	+	ND	–	+	–	–	+	ND	–	94/mL	95 pg/ml	+
Case 2	Kamada et al.	28/M	+	+	+	–	+	–	+	+	–	52/mL	Negative	+
Case 3	Sawada et al.	38/M	+	+	–	–	–	–	+	+	-	338/mL	69 pg/ml	+
Case 4	Katsuse et al.	44/F	+	+	+	–	–	–	+	ND	+	21/mL	Negative	+
Case 5	Fujimori et al.	46/M	+	+	+	+	+	+	+	+	+	56/mL	Negative	+
Case 6	Wang et al.	18/F	+	ND	ND	ND	ND	+	+	ND	+	2/mL	ND	ND

*ND, not described; Gd, Gadolinium; CSF, cerebrospinal fluid; MBP, myelin basic protein*.

CSF pleocytosis was observed in 5/6 patients, while CSF myelin basic protein (MBP) levels (<102 pg/ml) were not elevated in any of the six patients. A favorable response to steroid treatment was observed for all patients. The clinical symptoms and brain MRI findings were partially improved only by the administration of antiepileptic drugs but relapsed later and were relieved after steroid treatment in cases 2 and 4.

**Figure 1:**
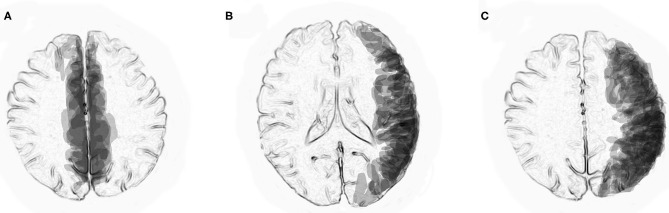
Superimposed MRI lesions from previously reported patients with anti-MOG antibody-associated unilateral cerebral cortical encephalitis (UCCE) and bilateral medial frontal cerebral cortical encephalitis (BFCCE). **(A)** Axial view at the level of the centrum semiovale indicates that MRI lesions in patients with anti-MOG antibody-associated BFCCE were mostly located in the peripheral territories of the anterior cerebral arteries. **(B,C)** Axial views at the level of the lateral ventricles and the centrum semiovale indicate that MRI lesions in patients with anti-MOG antibody-associated UCCE were mostly located in the peripheral territories of the middle cerebral arteries.

Superimposed MRI lesions in previously reported six patients with anti-MOG antibody-associated BFCCE (10, 16, 19–21) indicated that most of the lesions were distributed in the peripheral territories of the anterior cerebral arteries (Figure 1A). In contrast, superimposed MRI lesions in previously reported 13 patients with anti-MOG antibody-associated UCCE (9, 11, 14–16, 22–25) indicated that most of the lesions were distributed in the peripheral territories of the middle cerebral arteries (Figures 1B,C).

## Discussion

In this study, we showed that adult-onset anti-MOG antibody-associated BFCCE is a characteristic phenotype of cerebral cortical encephalitis. Similar to anti-MOG antibody-associated UCCE, BFCCE was characterized by an adult onset, main symptoms of headache, fever, and seizure, steroid responsiveness, and cerebral cortical FLAIR hyperintense lesions on MRI. In contrast, anti-MOG antibody-associated BFCCE presented with several characteristic clinical symptoms, such as paraparesis and lethargy, that are caused by the involvement of the motor cortex and medial side of the frontal lobe.

Paraparesis was observed in two patients (Cases 1 and 5). In case 1, paraparesis was observed only subjectively, and mild muscular weakness was objectively observed in the right lower limb. In case 5, the patient initially complained of dizziness and experienced a focal motor seizure in the right leg that subsequently generalized. Thereafter, the paraparesis progressed gradually, and the patient became completely paraplegic with spasticity. Therefore, paraparesis might be subtle or noticed as dizziness in the early stage of this illness. Furthermore, paraparesis without sensory symptoms might indicate cerebral cortical lesions. In contrast, in case 4, postictal paraparesis was observed due to epilepsia partialis continua.

To date, pathological studies on anti-MOG antibody-associated disease have revealed perivenous inflammatory demyelination (15, 26) with MOG-dominant myelin loss (13, 17). Furthermore, a recent pathological study that analyzed 2 autopsies and 22 brain biopsies reported that anti-MOG antibody-associated disorder was characterized by the coexistence of both perivenous and confluent white matter demyelination, with an overrepresentation of intracortical demyelinated lesions (27). Cortical demyelination was associated with meningeal inflammation, and in contrast to multiple sclerosis, intracortical rather than leukocortical demyelinated lesions predominated (27). These results may suggest that perivenous inflammatory demyelination in the cerebral cortex can be a primary pathological change in anti-MOG antibody-associated cerebral cortical encephalitis.

Although we considered that UCCE and BFCCE with anti-MOG antibodies would share a common pathogenesis, the factors that determine their unique lesion distribution have not been clarified. Interestingly, our study indicated that in typical patients with UCCE and BFCCE with anti-MOG antibodies, their lesion distribution mostly coincides with the peripheral territories of the middle cerebral arteries (MCAs) (9, 11, 14–16, 22–25) and anterior cerebral arteries (ACAs) (10, 16, 19–21), respectively. Moreover, vasodilatation of the branches of the MCAs or ACAs has been previously described in patients with anti-MOG antibody-associated UCCE or BFCCE (21, 22), although the vasodilatation observed in these patients might be explained by seizure activity. In contrast, we recently reported an adult case of anti-MOG antibody-associated left UCCE in which the patient only presented with ipsilateral headache without seizures, while MR angiography (MRA) showed dilatation of the left MCA branches that resolved after steroid treatment (11). These observations of the lesion distribution and vasodilatation might suggest that the peripheral vessels of the MCA or ACA are involved during lesion development.

Vascular involvement has also been suggested in other forms of anti-MOG antibody-associated encephalitis. For example, in anti-MOG-associated leukoencephalopathy and brainstem encephalitis, punctate and curvilinear gadolinium enhancement (PCGE) has been reported (28–30). Cerebral PCGE with hyperintensities on T2WI and FLAIR in the corresponding areas is an MRI finding that may be seen when the blood-brain barrier (BBB) of the small vessels is disrupted by a direct injury of the endothelial cells or by an angiocentric cellular infiltrate (31). However, since there have been other reports describing cortical lesions other than those observed in UCCE or BFCCE with anti-MOG antibodies (17), our hypothesis cannot be applied to all cases of anti-MOG antibody-associated cerebral cortical encephalitis. Moreover, vasodilatation must be caused secondarily by cortical inflammation.

Regarding the CSF findings, CSF pleocytosis was observed in most cases, while CSF MBP elevation was not observed, as has also been reported in cases of anti-MOG antibody-associated UCCE (9, 14). However, in general, CSF MBP levels can reflect myelin damage in the central nervous system, and pathological studies show demyelinating lesions in anti-MOG antibody-associated disorders. Furthermore, previous studies have indicated that CSF MBP levels are elevated in anti-MOG antibody-positive patients with neuromyelitis optica spectrum disorder (NMOSD), transverse myelitis, and acute disseminated encephalomyelitis (ADEM) (32). Therefore, the reason why CSF MBP elevation is rarely observed in patients with anti-MOG antibody-associated cerebral cortical encephalitis needs to be clarified in the future.

Last, our study has several limitations. First, we were only able to identify a small number of Asian cases. Second, we collected data retrospectively and could not obtain enough imaging information from non-case reports. Third, we are missing cases reported in journals other than CENI that are not searchable in PubMed.

## Conclusion

BFCCE with anti-MOG antibody presents characteristic clinical findings, such as paraparesis, lethargy, and memory decline, and radiological findings. Although ACA involvement might be observed in this disease entity, further analysis is needed to clarify its pathogenesis.

## Author Contributions

JF drafted the manuscript. MN and TY built figures and tables. IN supervised the study process. All authors contributed to the data review, interpretation of results, critically revised the manuscript, and approved the final version of the article.

## Conflict of Interest

IN is serving on scientific advisory boards for Biogen Japan and Novartis Pharma and is receiving honoraria for speaking engagements with Biogen Japan, Mitsubishi Tanabe Pharma, Novartis Pharma, Takeda Pharmaceutical, and Eisai. The remaining authors declare that the research was conducted in the absence of any commercial or financial relationships that could be construed as a potential conflict of interest. The reviewer SN declared a shared affiliation, with no collaboration, with the authors to the handling Editor.

## References

[1] Rostasy K Mader S Schanda K Huppke P Gartner J Kraus V. Anti-myelin oligodendrocyte glycoprotein antibodies in pediatric patients with optic neuritis. Arch Neurol. (2012) 69:752–6. 10.1001/archneurol.20112956 22371853

[2] Sato DK Callegaro D Lana-Peixoto MA Waters PJ de Haidar Jorge FM Takahashi T. Distinction between MOG antibody-positive and AQP4 antibody-positive NMO spectrum disorders. Neurology. (2014) 82:474–81. 10.1212/WNL0000000000000101 24415568 PMC3937859

[3] Kitley J Woodhall M Waters P Leite MI Devenney E Craig J. Myelin-oligodendrocyte glycoprotein antibodies in adults with a neuromyelitis optica phenotype. Neurology. (2012) 79:1273–7. 10.1212/WNL0b013e31826aac4e 22914827

[4] Reindl M Pauli Di F Rostasy K Berger T. The spectrum of MOG autoantibody-associated demyelinating diseases. Nat Rev Neurol. (2013) 9:455–61. 10.1038/nrneurol.2013118 23797245

[5] Fujihara K Sato DK Nakashima I Takahashi T Kaneko K Ogawa R Myelin oligodendrocyte glycoprotein immunoglobulin G-associated disease: An overview. Clin Exp Neuroimmunol. (2018) 9:48–55. 10.1111/cen312434

[6] Fujimori J Takahashi T Matsumoto Y Fujihara K Takai Y Misu T. Two Japanese cases of anti-MOG antibody-associated encephalitis that mimicked neuro-Behcet's disease. J Neuroimmunol. (2019) 334:577002. 10.1016/j.jneuroim.2019577002 31279093

[7] Fujimori J Nakashima I Linear pontine trigeminal root lesion in a patient with anti-myelin oligodendrocyte glycoprotein antibody-associated encephalitis. Clin Exp Neuroimmunol. (2020) 11:122–5. 10.1111/cen312555

[8] Fujimori J Nakashima I Cerebellar lesions in a patient with anti-myelin oligodendrocyte glycoprotein antibody-associated encephalitis. Clin Exp Neuroimmunol. (2019) 10:264–6. 10.1111/cen312539

[9] Ogawa R Nakashima I Takahashi T Kaneko K Akaishi T Takai Y. MOG antibody-positive, benign, unilateral, cerebral cortical encephalitis with epilepsy. Neurol Neuroimmunol Neuroinflamm. (2017) 4:e322. 10.1212/NXI0000000000000322 28105459 PMC5241006

[10] Fujimori J Takai Y Nakashima I Sato DK Takahashi T Kaneko K. Bilateral frontal cortex encephalitis and paraparesis in a patient with anti-MOG antibodies. J Neurol Neurosurg Psychiatry. (2017) 88:534–6. 10.1136/jnnp-2016-315094 28209651

[11] Fujimori J Ogawa R Murata T Jin K Yazawa Y Nakashima I. Unilateral chronic pulsatile headache as the single manifestation of anti-MOG antibody-associated unilateral cerebral cortical encephalitis. J Neuroimmunol. (2020) 346:577322. 10.1016/j.jneuroim.2020577322 32682139

[12] Dos Passos GR Oliveira LM da Costa BK Apostolos-Pereira SL Callegaro D Fujihara K. MOG-IgG-associated optic neuritis, encephalitis, and myelitis: lessons learned from neuromyelitis optica spectrum disorder. Front Neurol. (2018) 9:217. 10.3389/fneur.2018.00217 29670575 PMC5893792

[13] Takai Y Misu T Kaneko K Chihara N Narikawa K Tsuchida S. Myelin oligodendrocyte glycoprotein antibody-associated disease: an immunopathological study. Brain. (2020) 143:1431–46. 10.1093/brain/awaa102 32412053

[14] Budhram A Mirian A Le C Hosseini-Moghaddam SM Sharma M Nicolle MW. Unilateral cortical FLAIR-hyperintense lesions in anti-MOG-associated encephalitis with seizures (FLAMES): characterization of a distinct clinico-radiographic syndrome. J Neurol. (2019) 266:2481–7. 10.1007/s00415-019-09440-8 31243540

[15] Patterson K Iglesias E Nasrallah M González-Álvarez V Suñol M Anton J. Anti-MOG encephalitis mimicking small vessel CNS vasculitis. Neurol Neuroimmunol Neuroinflamm. (2019) 6:e538. 10.1212/NXI0000000000000538 30800721 PMC6384022

[16] Wang L ZhangBao J Zhou L Zhang Y Li H Li Y. Encephalitis is an important clinical component of myelin oligodendrocyte glycoprotein antibody associated demyelination: a single-center cohort study in Shanghai, China. Eur J Neurol. (2019) 26:168–74. 10.1111/ene13790 30133068

[17] Ikeda T Yamada K Ogawa R Takai Y Kaneko K Misu T. The pathological features of MOG antibody-positive cerebral cortical encephalitis as a new spectrum associated with MOG antibodies: a case report. J Neurol Sci. (2018) 392:113–5. 10.1016/j.jns.2018.06028 30048831

[18] Cobo-Calvo A Ruiz A Maillart E Audoin B Zephir H Bourre B. Clinical spectrum and prognostic value of CNS MOG autoimmunity in adults: The MOGADOR study. Neurology. (2018) 90:e1858–69. 10.1212/WNL0000000000005560 29695592

[19] Kamada T Miura S Harada M Irie A Kikuchi S Taniwaki T. Bilateral cingulate cortices lesions in two autoantibodies directed against MOG (MOG-Ab)-positive patients. Multiple Scler Relat Disord. (2019) 29:108–10. 10.1016/j.msard.2019.01035 30708307

[20] Sawada J Katayama T Toyoshima S Nitamizu S Yamamoto K Fukuura A Three Japanese adult cases of brain lesions with anti-myelin oligodendrocyte glycoprotein antibodies lacking optic neuritis and myelitis. Clin Exp Neuroimmunol. (2018) 9:162–8. 10.1111/cen312459

[21] Katsuse K Shimizu G Saito Sato N Hatano K Yagi S Kimura T. Epilepsia partialis continua as an early sign of anti-myelin oligodendrocyte glycoprotein antibody-positive encephalitis. Int Med. (2020) 59:1445–9. 10.2169/internalmedicine3076-19 32132331 PMC7332626

[22] Adachi H Ide Y Takahashi T Yoneda Y Kageyama Y. Cerebral cortical encephalitis with anti-myelin oligodendrocyte glycoprotein (MOG) antibody. Clin Neurol. (2018) 58:767–70. 10.5692/clinicalneurolcn-001224 30487364

[23] Sugimoto T Ishibashi H Hayashi M Tachiyama K Fujii H Kaneko K. A case of anti-MOG antibody-positive unilaterally dominant meningoencephalitis followed by longitudinally extensive transverse myelitis. Multiple Scler Relat Disord. (2018) 25:128–30. 10.1016/j.msard.2018.07028 30071506

[24] Fukushima N Suzuki M Ogawa R Hayashi K Takanashi JI Ohashi T. A case of anti-MOG antibody-positive multiphasic disseminated encephalomyelitis co-occurring with unilateral cerebral cortical encephalitis. Clin Neurol. (2017) 57:723–8. 10.5692/clinicalneurolcn-001078 29070756

[25] Yamamoto D Uchiyama T Ohashi T Iizuka T Case of steroid-responsive unilateral encephalitis with anti-myelin oligodendrocyte glycoprotein antibodies. Neurol Clin Neurosci. (2017) 5:101–2. 10.1111/ncn312119

[26] Papathanasiou A Tanasescu R Davis J Rocha MF Singhal S O'Donoghue MF. MOG-IgG-associated demyelination: focus on atypical features, brain histopathology and concomitant autoimmunity. J Neurol. (2020) 267:359–68. 10.1007/s00415-019-09586-5 31641876

[27] Höftberger R Guo Y Flanagan EP Lopez-Chiriboga AS Endmayr V Hochmeister S. The pathology of central nervous system inflammatory demyelinating disease accompanying myelin oligodendrocyte glycoprotein autoantibody. Acta Neuropathol. (2020) 139:875–92. 10.1007/s00401-020-02132-y 32048003 PMC7181560

[28] Komatsu T Matsushima S Kaneko K Fukuda T. Perivascular enhancement in anti-MOG antibody demyelinating disease of the CNS. J Neurol Neurosurg Psychiatry. (2019) 90:111–2. 10.1136/jnnp-2018-319235 30297517

[29] Berzero G Taieb G Marignier R Younan N Savatovsky J Leclercq D. CLIPPERS mimickers: relapsing brainstem encephalitis associated with anti-MOG antibodies. Eur J Neurol. (2018) 25:e16–7. 10.1111/ene13483 29356261

[30] Symmonds M Waters PJ Küker W Leite MI Schulz UG. Anti-MOG antibodies with longitudinally extensive transverse myelitis preceded by CLIPPERS. Neurology. (2015) 84:1177–9. 10.1212/WNL0000000000001370 25681455 PMC4371412

[31] Taieb G Duran-Pena A de Chamfleur NM Moulignier A Thouvenot E Allou T. Punctate and curvilinear gadolinium enhancing lesions in the brain: a practical approach. Neuroradiology. (2016) 58:221–35. 10.1007/s00234-015-1629-y 26700824

[32] Kaneko K Sato DK Nakashima I Nishiyama S Tanaka S Marignier R. Myelin injury without astrocytopathy in neuroinflammatory disorders with MOG antibodies. J Neurol Neurosurg Psychiatry. (2016) 87:1257–9. 10.1136/jnnp-2015-312676 26800711

